# Genetic Diversity and New Lineages of Dengue Virus Serotypes 3 and 4 in Returning Travelers, Germany, 2006–2015

**DOI:** 10.3201/eid2302.160751

**Published:** 2017-02

**Authors:** Sami Shihada, Petra Emmerich, Corinna Thomé-Bolduan, Stephanie Jansen, Stephan Günther, Christina Frank, Jonas Schmidt-Chanasit, Daniel Cadar

**Affiliations:** Bernhard Nocht Institute for Tropical Medicine, Hamburg, Germany (S. Shihada, P. Emmerich, C. Thomé-Bolduan, S. Jansen, S. Günther, J. Schmidt-Chanasit, D. Cadar);; Robert Koch Institute, Berlin, Germany (C. Frank);; German Centre for Infection Research, Hamburg (J. Schmidt-Chanasit)

**Keywords:** dengue virus, DENV, lineage, traveler, travel-associated infection, phylogeny, Germany, serotype, viruses, vector-borne, mosquito-borne

## Abstract

During 2006–2015, we analyzed 70 dengue virus (DENV) strains isolated from febrile travelers returning to Germany. High genetic diversity, including multiple co-circulating DENV lineages and emerging new lineages of DENV-3 and DENV-4, was demonstrated. Our passive surveillance system based on returning travelers yielded substantial information on DENV diversity.

Although dengue virus (DENV) infects ≈390 million persons annually and one third of the world’s population is at risk for infection, there is no effective vaccine or specific antiviral therapy for infection with DENV (*1*). Dengue is a rapidly spreading mosquito-borne viral disease and the most frequent cause of febrile illness among international travelers returning from DENV-endemic tropical areas, such as Southeast Asia, the western Pacific region, and the Americas (*2*,*3*). Viremic travelers have the potential to introduce DENV into DENV-free or nonendemic areas where competent mosquito vectors are present (*4*). Reintroduction of DENV in regions that had been considered free of the disease for many years has also been observed (*5*–*7*).

Phylogenetic analysis has elucidated the origins, epidemiology, and forces that shape DENV molecular evolution in nature (*8*). For example, according to official German air travel statistics reports, 4,855,763 air trips were taken in 2011 from Germany to countries listed as DENV-endemic areas by the World Health Organization; 10%–20% each of these trips were made to India, Thailand, and Brazil; and 5%–10% each flew to Singapore, Mexico, and the Dominican Republic (*9*). We determined the genetic relatedness and molecular epidemiology of DENV isolates from travelers returning to Germany during 2006–2015.

## The Study

During 2006–2015, we analyzed 15,876 acute-phase serum samples from patients with suspected DENV infection; the samples had been submitted to the World Health Organization Collaborating Centre for Arbovirus and Hemorrhagic Fever Reference and Research for diagnostic testing. We tested all samples by using DENV type-specific real-time reverse transcription PCR (rRT-PCR) (*10*) or an antigen-capture ELISA (Platelia Dengue NS1 Ag; Bio-Rad, Hercules, CA, USA) and in-house DENV IgG and IgM indirect immunofluorescence assays. rRT-PCR– and nonstructural protein 1–positive serum samples that tested negative for DENV IgG and IgM were spread onto Vero E6 cells and incubated for 7 days at 37°C; successful DENV isolation was identified by rRT-PCR. We extracted viral RNA from cell culture supernatants by using the QIAamp Viral RNA Mini Kit (QIAGEN, Hilden, Germany). 

We successfully isolated 70 DENV strains originating from 20 countries (Technical Appendix). We amplified the complete envelope glycoprotein (E) gene using DENV type–specific degenerate primers (Technical Appendix). Sequence assembly, analysis, and multiple alignments were performed with Geneious version 7.1.8 (Biomatters, Auckland, New Zealand). All available complete envelope gene sequences of DENV serotypes 1–4 (DENV-1–4), except laboratory strains and potential recombinants, were retrieved from GenBank and compared with those sequenced in this study. The phylogenetic relationships and origin of the imported DENV isolates were analyzed by the maximum likelihood method in the RAxML program (*11*) with general time-reversible plus gamma distribution substitution model and a rapid bootstrap (100 replicates) procedure, and visualized in FigTree version 1.4.3 (http://tree.bio.ed.ac.uk/software/figtree/). 

**Figure 1:**
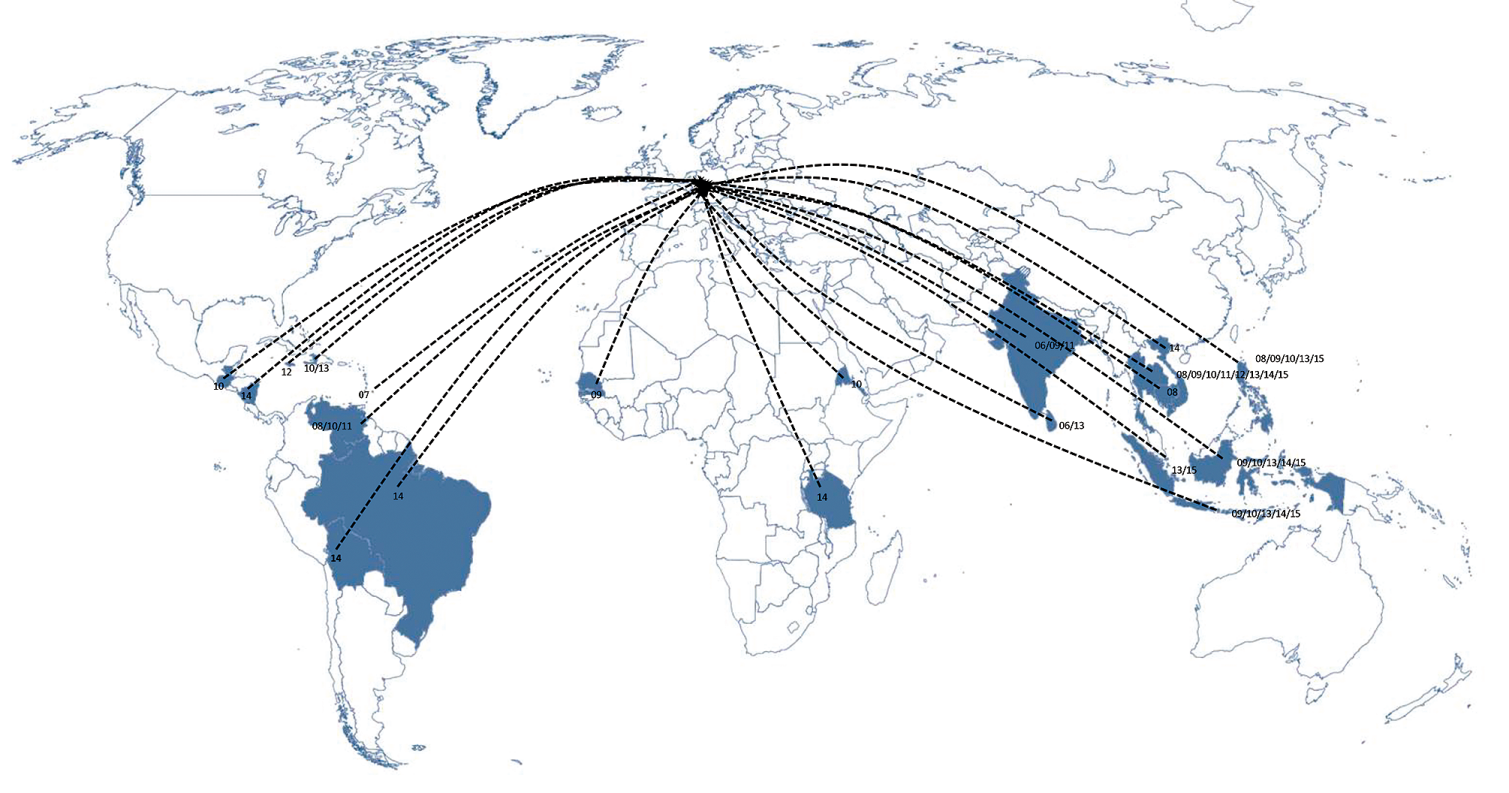
Geographic origin of dengue viruses isolated from travelers returning to Germany, 2006–2015.

**Figure 2:**
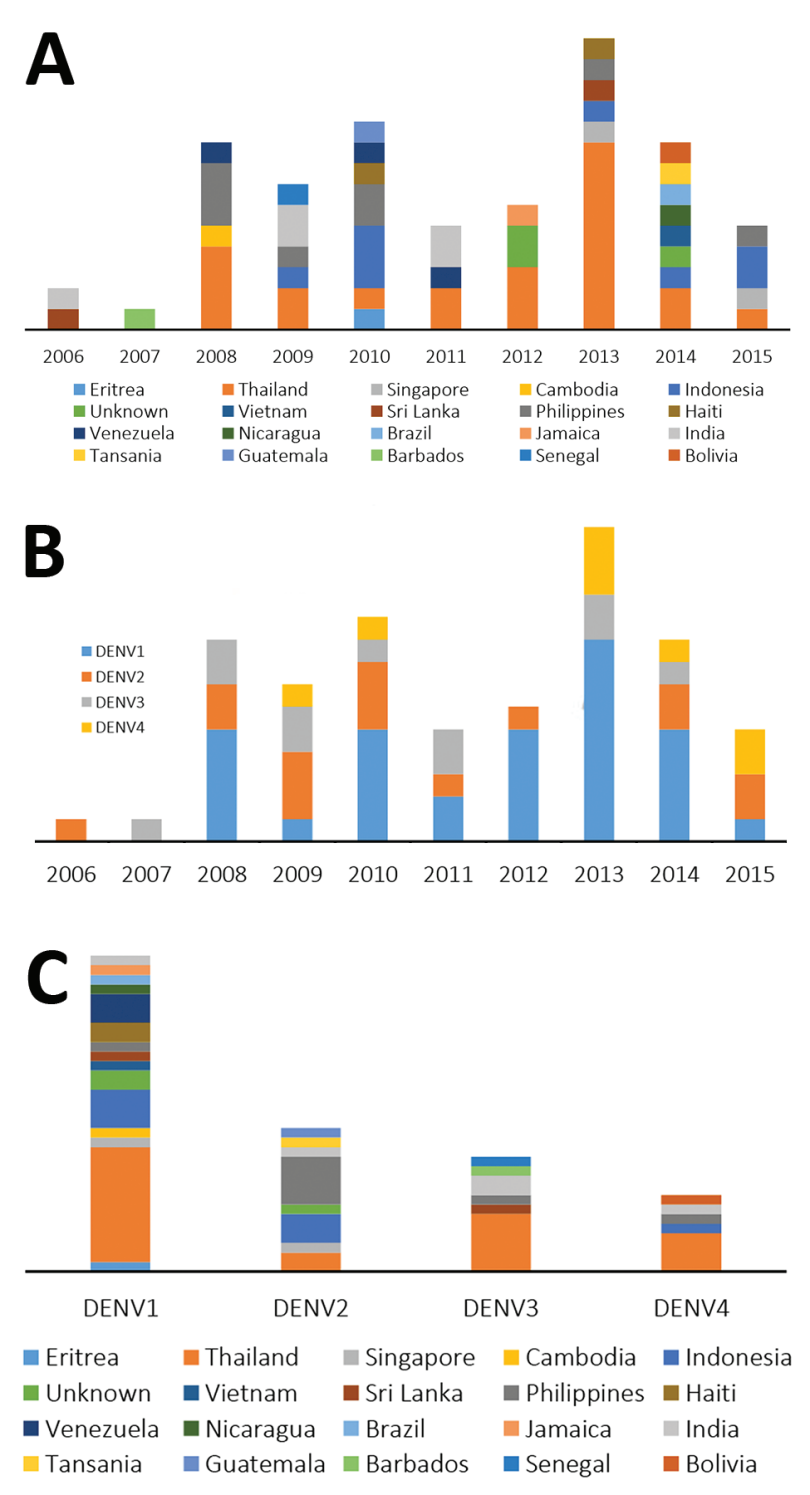
Year of introduction (A) and diversity (B and C) of dengue viruses isolated from travelers returning to Germany, 2006–2015.

Most DENV infections were acquired in Thailand (35.7%), followed by Indonesia (12.8%), the Philippines (10%), and India (7.1%). The proportion of cases imported from other countries in Asia, Africa, and the Americas ranged between 1.4% and 4.3% (Figures 1,2). Phylogenetic analysis revealed a high genetic diversity of DENV-1–4 in travelers from Germany, including co-circulation of multiple genetically diverse viral lineages that were closely related to those previously circulating in the Americas and Southeast Asia (mostly Thailand, Indonesia, and the Philippines) (Technical Appendix). DENV-1 was the predominant and most genetically diverse type, representing 33 sequences and clustering into 23 phylogenetically distinct lineages. Most of the isolates belonged to genotypes I and II, which are circulating in Southeast Asia (*12*). These genotypes represent dominant regional variants; only a few strains are closely related to viruses circulating in the Americas (genotype V, lineages 3–6) or to a genotype III virus in India. All but 3 DENV-2 isolates were the Cosmopolitan genotype, and the source population was limited mostly to countries in Southeast Asia (Technical Appendix). 

DENV-2 phylogeny provides evidence for 7 phylogenetically distinct introductions of the virus into travelers from Germany; the origins of these isolates were predominantly in Bali and the Philippines. DENV-3 sequences belonged to genotypes I–III; these isolates were closely related to lineages representing regional or local variants, except lineages 8 and 9 (Technical Appendix).

A key phylogenetic pattern was the presence of a new lineage, L6 (Technical Appendix), within DENV-3 phylogeny comprising closely related isolates sampled from German, Taiwanese, and Chinese travelers returning from Thailand and Laos during 2012–2014. A similar pattern of clustering to that for DENV-3 was observed for DENV-4 isolates. Thus, most DENV-4 from travelers were infected with genotype I and III strains closely related to lineages commonly sampled within Southeast Asia and to a genotype II isolate from Bolivia. A key phylogenetic pattern was the presence of a new lineage, L3, within DENV-4 phylogeny (Technical Appendix) with sequences found in samples from patients tested during 2013–2015 with travel histories to Thailand, China, and the Philippines (lineage 3). Lineage 3 was the most recently circulating lineage detected in this study. This clustering is compatible with extensive viral traffic between Thailand, China, and the Philippines; Thailand, where most of the DENV infections were acquired, is thus a possible source of a virus population responsible for local or regional outbreaks.

## Conclusions

Countries in Southeast Asia that are considered DENV hyperendemic are increasingly popular tourist destinations for residents of Germany; thus, German travelers to these countries are potentially exposed to multiple types and genotypes of DENV. A high prevalence of DENV infection has been reported in travelers returning from DENV-endemic areas, emphasizing the importance of international travelers as potential sources of imported disease or sentinels for local outbreaks in DENV-free or non–DENV-endemic areas (*2*,*3*). The relative risk of infection by country is difficult to calculate without attention to seasonal fluctuations in dengue fever incidence and travel patterns. Broadly speaking, though, among the top contributing countries mentioned, the comparative risk of infection with travel-associated DENV appears much higher in the Philippines and Indonesia (10% and 12.8% of the cases, compared with 1.7% and 2.8%, respectively, of travelers from Germany to DENV-endemic countries) than in Thailand (35.7% of the cases versus 15.0% of the travelers) and the lowest in India (7.1% of the cases versus 18.5% of the travelers). 

We investigated DENV diversity and origin of infection in travelers returning to Germany from DENV-endemic areas and identified a high genetic diversity of DENV genotypes and lineages. Notably, 2 of these lineages (DENV-3, genotype III, lineage 6 and DENV-4, genotype III, lineage 6) appear to have emerged very recently and are still responsible for local outbreaks in countries in Southeast Asia, thus reiterating the need to monitor the appearance and spread of novel lineages. Most investigated isolates were closely related to lineages known to have circulated in Thailand, the Philippines, and Indonesia, indicating that these countries serve as a major source of multiple DENV lineages (*12*). The observed high numbers of co-circulating lineages in the Thai, Indonesian, and Philippines source populations support the hypothesis of multiple geographic origins or extensive virus interchange among these countries (Technical Appendix). 

Surveillance of symptomatic returned travelers can provide information on circulating DENV genotypes and lineages in heavily visited tourist areas and DENV-endemic regions. In Europe, the emergence of arboviruses should be particularly monitored because of the introduction and expansion of the DENV vector *Aedes albopictus* mosquito. In Germany, where recently introduced *Ae. albopictus* mosquitos have spread in the southwestern part of the country, international travelers and the presence of competent vectors could potentially facilitate seasonal local transmission of DENV (*7*,*13*,*14*). Our findings indicate a diverse array of imported DENV infections in travelers from Germany and emphasize the need for a continued surveillance of DENV infections in non–DENV-endemic regions as well as prompt and rapid serologic and molecular testing for DENV infection in febrile patients returning from DENV-endemic countries.

Technical Appendix Primers used for the amplification and sequencing of the complete envelope gene of dengue virus types and origin and genetic relatedness of dengue viruses entering Germany; figure depicting phylogenetic relationships of DENV strains isolated from travelers returning to Germany, 2006–2015.
