# Supplementary material for: Genetic Diversity and New Lineages of Dengue Virus Serotypes 3 and 4 in Returning Travelers, Germany, 2006–2015
Source: Emerg Infect Dis. 2017 Feb;23(2):272–5. doi: 10.3201/eid2302.160751 (PMC5324807; doi:10.3201/eid2302.160751)
Supplement: Technical Appendix — Primers used for the amplification and sequencing of the complete envelope gene of dengue virus types and origin and genetic relatedness of dengue viruses entering Germany; figure depicting phylogenetic relationships of DENV strains isolated from travelers returning to Germany, 2006–2015. [file 16-0751-Techapp-s1.pdf]

Article DOI: http://dx.doi.org/10.3201/eid2302.160751

Genetic Diversity and New Lineages of Dengue Virus Types 3 and 4 in Travelers Returning to Germany, 2006–2015

Technical Appendix

Technical Appendix Table 1. Primers used for the amplification and sequencing of complete envelope gene of dengue virus types

Primer name/serotype	Primer sequence, 5′→3′	Position and orientation*	Length, bp
PANENV-1F-DENV-1	ASA CRT GGG TGA CYT ATG GVA	557–577 F	1071
PANENV-1R-DENV-1	CAG TRT GCA TYG CTC CTT CYT	1607–1627 R	
PANENV-2F-DENV-1	CAG CYC ATG CDA ARA AVC AGG	1565–1585 F	858
PANENV-2R-DENV-1	TGT ATT GCT CTG TCC ARG TGT	2402–2422 R	
PANENV-1F-DENV-2	TCG CTC YTT CAA TGR CRA TGC	824–844 F	912
PANENV-1R-DENV-2	ATG ACA TTC CTT TRA GYT GT	1716–1735 R	
PANENV-2F-DENV-2	GGD TCY CAA GAD GGG GCY ATG	1600–1620 F	821
PANENV-2R-DENV-2	TAY TGY TCT GTC CAY GTR TGY	2400–2420 R	
PANENV-1F-DENV-3	CAC GYR CYC ARA CYT GGA TGT	656–676 F	656
PANENV-1R-DENV-3	ATT GCT CCY TCT TGI GAY CCA	1593–1613 R	
PANENV-2F-DENV-3	CCA TGG RCA TCA GGR GCI AYA	1489–1509 F	868
PANENV-2R-DENV-3	GTT CTT TMC CRT TCC ART TTA	2336–2356 R	
PANENV-1F-DENV-4	CCA TCY TAY GGA ATG CGI TGC	826–846 F	782
PANENV-1R-DENV-4	TCY TGA GAY CCR ARY ACT GTC	1597–1607 R	
PANENV-2F-DENV-4	CCT CAT GCC AAG AGA CAG GAT	1564–1584 F	784
PANENV2R-DENV-4	TCC ATG ACA CCA CAC ARC CC	2328–2347 R	

*Nucleotide positions are according to the genomes for DENV-1 (GenBank accession no. KM204119), DENV-2 (GenBank accession no. JX66948), DENV-3 (GenBank accession no. EU482596), and DENV-4 (GenBank accession no. EU854301). DENV, dengue virus; F, forward orientation; R, reverse orientation.

Technical Appendix Table 2. Origin and genetic relatedness of dengue viruses isolated from travelers returning to Germany, 2006–2015*

Sequence ID/GenBank accession no.	Travel history	Year of collection	Type	Genotype/lineage
2522/KU509258	Eritrea	2010	DENV-1	II/L9
2878/KU509259	Ko Samui, Thailand	2008	DENV-1	I/L10
2334/KU509313	Singapore	2013	DENV-1	I/L12
18439/KU509265	NA	2012	DENV-1	I/L11
3616/KU509260	Cambodia	2008	DENV-1	I/L10
18014/KU509263	Thailand	2012	DENV-1	I/L16

Sequence ID/GenBank accession no.	Travel history	Year of collection	Type	Genotype/lineage
18805/KU509266	Thailand	2012	DENV-1	I/L16
8902/KU509289	Bali, Indonesia	2015	DENV-1	I/L23
9328/KU509291	Thailand	2013	DENV-1	I/L21
24278/KU509309	Bali, Indonesia	2013	DENV-1	I/L19
11656/KU509293	Thailand	2014	DENV-1	I/L18
1763/KU509314	Thailand	2013	DENV-1	I/L17
8356/KU509253	Bali, Indonesia	2014	DENV-1	I/L20
11100/KU509292	Vietnam	2014	DENV-1	I/L22
1990/KU509257	Thailand	2008	DENV-1	I/L18
8915/KU509290	Thailand	2013	DENV-1	I/L13
12563/KU509294	Thailand	2014	DENV-1	I/L13
23789/KU509310	Sri Lanka	2013	DENV-1	I/L15
17461/KU509250	Thailand	2012	DENV-1	I/L14
384/KU509256	Thailand	2009	DENV-1	I/L10
3845/KU509262	Thailand	2008	DENV-1	I/L10
00808/KU509316	Thailand	2013	DENV-1	II/L7
1301/KU509315	Philippines	2013	DENV-1	II/L7
3746/KU509261	Bali, Indonesia	2010	DENV-1	II/L8
23444/KU509295	Haiti	2013	DENV-1	III/L5
3852/KU509251	Margarita Island, Venezuela	2008	DENV-1	III/L4
4876/KU509252	Venezuela	2010	DENV-1	III/L4
10429/KU509254	Aragua, Venezuela	2011	DENV-1	III/L3
18037/KU509264	Haiti	2010	DENV-1	III/L5
5353/KU509312	Nicaragua	2014	DENV-1	III/L6
8246/KU509311	Brazil	2014	DENV-1	III/L2
17388/KU509249	Jamaica	2012	DENV-1	III/L5
16687/KU509255	India	2011	DENV-1	III/L1
1365/KU509272	Thailand	2009	DENV-2	Cosmopolitan/L10
3849/KU509275	Philippines	2008	DENV-2	Cosmopolitan/L4
3850/KU509276	Philippines	2008	DENV-2	Cosmopolitan/L4
14706/KU509277	Philippines	2010	DENV-2	Cosmopolitan/L4
3519/KU509274	Philippines	2010	DENV-2	Cosmopolitan/L3
973/KU509269	Philippines	2009	DENV-2	Cosmopolitan/L5
979/KU509270	Bali, Indonesia	2012	DENV-2	Cosmopolitan/L6
4584/KU509308	Bali, Indonesia	2015	DENV-2	Cosmopolitan/L6
6478/KU509307	Tanzania	2014	DENV-2	Cosmopolitan/L7
671/KU509268	Bali, Indonesia	2009	DENV-2	Cosmopolitan/L9
017079/KU509305	Bali, Indonesia	2014	DENV-2	Cosmopolitan/L8
2627/KU509271	India	2006	DENV-2	Cosmopolitan/L2
15766/KU509306	Singapore	2015	DENV-2	Cosmopolitan/L1
3229/KU509273	Thailand	2011	DENV-2	Asian 1/L12
30/KU509267	Guatemala	2010	DENV-2	Asian 2/L13
4397/KX147335	Singapore	2015	DENV-2	Asian 1/L11
254/KU509278	Barbados	2007	DENV-3	III/L5
632/KU509279	Philippines	2008	DENV-3	I/L1
1631/KU509280	Ko Samui, Thailand	2011	DENV-3	II/L3
2994/KU509281	India	2009	DENV-3	III/L7
3140/KU509282	Senegal	2009	DENV-3	III.L9
3404/KU509283	Sri Lanka	2006	DENV-3	III/L4
3847/KU509284	Thailand	2008	DENV-3	II/L3
8561/KU509285	Thailand	2010	DENV-3	I/L2
9468/KU509286	India	2011	DENV-3	III/L8
10803/KU509302	Thailand	2013	DENV-3	III/L6
10407/KU509303	Thailand	2013	DENV-3	III/L6
9846/KU509304	Thailand	2014	DENV-3	III/L6
3274/KU509287	India	2009	DENV-4	I/L1
6120/KU509288	Bali, Indonesia	2010	DENV-4	III/L4
22712/KU509296	Thailand	2013	DENV-4	I/L2
15983/KU509297	Philippines	2015	DENV-4	III/L3
12489/KU509298	Thailand	2015	DENV-4	III/L3
3186/KU509299	Thailand	2013	DENV-4	III/L3
3060/KU509300	Thailand	2013	DENV-4	I/L2
0831/KU509301	Bolivia	2014	DENV-4	II/L5
8635/KX147336	Thailand	2014	DENV-4	III/L3

*The isolates represent cases investigated by the World Health Organization Collaborating Centre for Arbovirus and Hemorrhagic Fever Reference and Research. DENV, dengue virus; NA, not applicable.

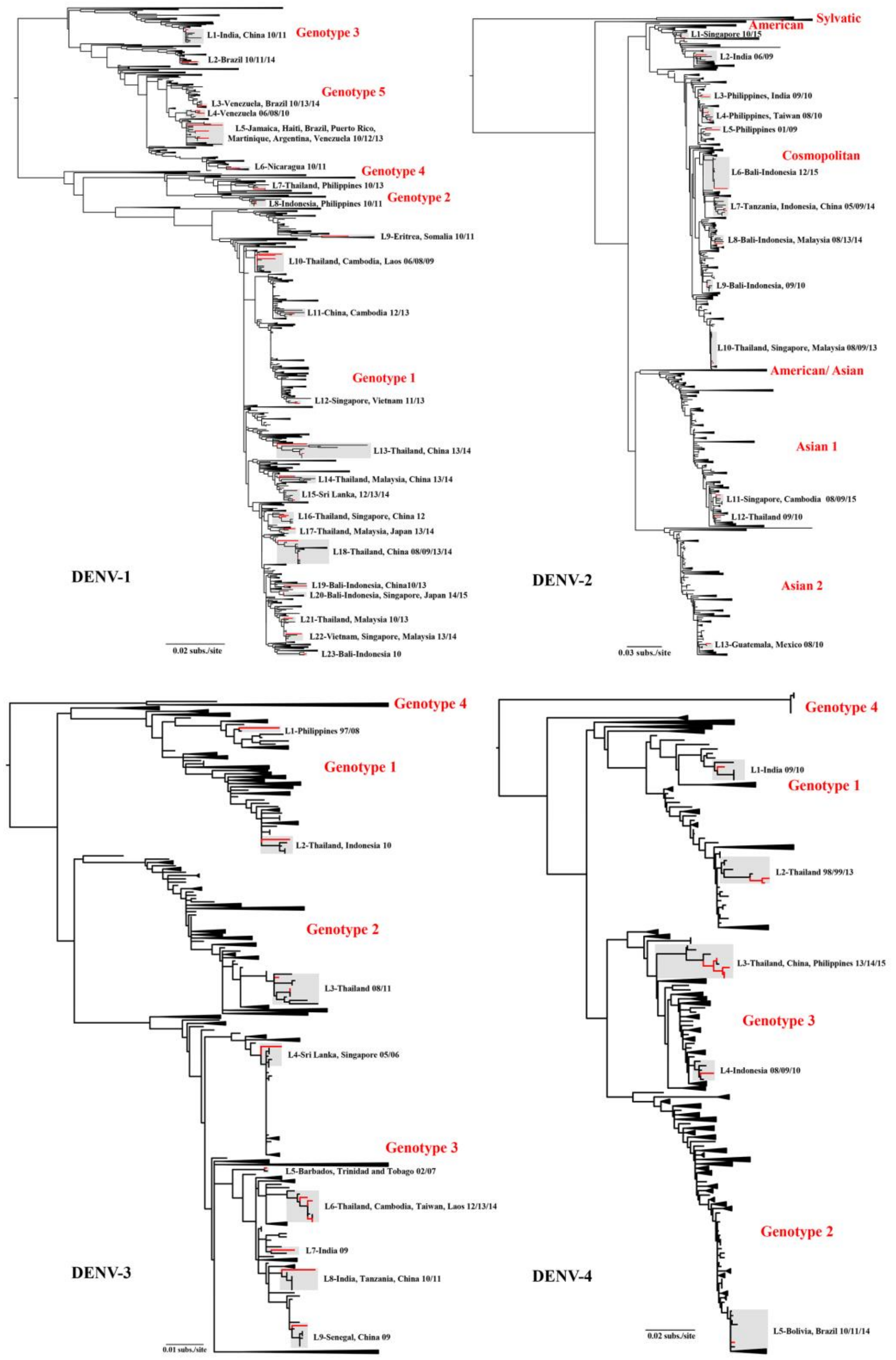

Technical Appendix Figure. Phylogenetic relationships, based on complete envelope gene sequences, of dengue virus (DENV) strains isolated from travelers returning to Germany, 2006–2015. For better visualization of the position of the sequences generated during this study, and because of the very large

number of sequences (DENV-1 = 3884 taxa; DENV-2 = 3498 taxa; DENV-3 = 1870 taxa; DENV-4 = 890 taxa) included, several phylogenetic clusters have been collapsed and depicted as triangles. The major genotypes, lineages (gray boxes) in which our sequences (strains sampled from German travelers colored in red) are grouped, and geographic origins of the members of the lineages, as well as the year of isolation, are indicated. Statistical support of grouping (maximum-likelihood bootstrap replicates $\geq$70%) is indicated with an asterisk. Scale bar indicates nucleotide substitution per site.
